# Tracking Dynamic Microvascular Changes during Healing after Complete Biopsy Punch on the Mouse Pinna Using Optical Microangiography

**DOI:** 10.1371/journal.pone.0057976

**Published:** 2013-02-28

**Authors:** Yeongri Jung, Suzan Dziennis, Zhongwei Zhi, Roberto Reif, Ying Zheng, Ruikang K. Wang

**Affiliations:** Department of Bioengineering, University of Washington, Seattle, Washington, United States of America; Okayama University, Japan

## Abstract

Optical microangiography (OMAG) and Doppler optical microangiography (DOMAG) are two non-invasive techniques capable of determining the tissue microstructural content, microvasculature angiography, and blood flow velocity and direction. These techniques were used to visualize the acute and chronic microvascular and tissue responses upon an injury *in vivo*. A tissue wound was induced using a 0.5 mm biopsy punch on a mouse pinna. The changes in the microangiography, blood flow velocity and direction were quantified for the acute (<30 min) wound response and the changes in the tissue structure and microangiography were determined for the chronic wound response (30 min–60 days). The initial wound triggered recruitment of peripheral capillaries, as well as redirection of main arterial and venous blood flow within 3 min. The complex vascular networks and new vessel formation were quantified during the chronic response using fractal dimension. The highest rate of wound closure occurred between days 8 and 22. The vessel tortuosity increased during this time suggesting angiogenesis. Taken together, these data signify that OMAG has the capability to track acute and chronic changes in blood flow, microangiography and structure during wound healing. The use of OMAG has great potential to improve our understanding of vascular and tissue responses to injury in order to develop more effective therapeutics.

## Introduction

Cutaneous wounds are common and include incisions/lacerations, abrasions, contusions, puncture wounds, burns and ulcers. Complications during the cutaneous wound healing process are a significant healthcare challenge and economic concern. Each year it is estimated that over one million people are treated for burn injury alone [1]. In addition to undesirable scar formation, skin wounds often become severely infected which can become fatal. Skin damage due to a wound, burn or illness may lead to major complications, such as diabetic ulcers, psoriasis and bed sores [1]–[3]. For example, approximately one third of patients with diabetes mellitus suffer from cutaneous manifestations of the disease including non-healing ulcers [4].

One of the main goals during tissue repair is to achieve rapid wound closure. Recent advances in cellular and molecular biology have determined that the basic biological processes involved in wound healing are inflammation, tissue formation and tissue remodeling [5], [6]. The vasculature must be a key player in promoting tissue survival and repair during these processes as it carries the tissue's blood supply and is the site for initiation of inflammatory events in the affected area. However, little is known about the changes which occur to the vasculature after tissue damage and during repair. To design effective treatments, further understanding of both vascular and tissue restoration during wound healing is required. Studies of wound healing mechanisms have been limited due to the lack of available technologies suitable to study real time vascular and tissue responses *in vivo*.

Most studies have been based on analyzing histological samples [5], which limits the understanding of the dynamic mechanisms such as compensatory changes in blood flow and subsequent angiogenesis with concomitant tissue remodeling. *In vitro* models are thus limited and cannot assess the physiologically relevant interrelationship between the vasculature and tissue which act in concert to promote repair. Conventional *in vivo* imaging techniques such as magnetic resonance imaging, and computed tomography angiography fail to provide sufficient spatial and temporal resolutions to measure rapid blood flow changes in small vessels down to the capillary level [7], [8]. Multiphoton microscopy is a high resolution imaging technique which allows for the visualization of capillaries; however, it has a small field of view, limited penetration depth, and requires the use of exogenous contrast agents [9]. High frequency ultrasound has relatively low spatial resolution (about 50 µm), can achieve depths of<3 mm and is sensitive to a minimum velocity of 0.2 mm/s [10], [11]. Photoacoustic microscopy is an imaging modality that can detect changes in the concentration of hemoglobin concentration in blood within small vessels [12], which has been previously used to determine the metabolic rate of oxygen [13]. However, photoacoustic microscopy requires contact with the tissue of interest, which may complicates the interpretation of the final results. Laser speckle imaging has also been used to investigate blood flow after biopsy punch in the mouse pinna [14]. This technique images the relative changes in blood flow but it is limited to large vessels and obtains a 2D map where the depth axis is roughly averaged [15], [16]. These drawbacks of current imaging modalities limit the detailed study of vascular changes and tissue repair *in vivo*. Therefore, a noninvasive method for accessing tissue and vessel regeneration with capillary resolution is needed to better monitor the vascular dynamics and tissue remodeling during the wound healing process.

Optical coherence tomography (OCT) is a non-invasive imaging technology capable of producing high-resolution cross-sectional images of tissue structures. By using a beam of near infrared light and measuring the back-scattered/back-reflected light, a cross-sectional image through the tissue can be rendered [17], [18]. Recent advances in OCT have been reported for high resolution imaging of cutaneous wounds to assess the structural characteristics associated with the healing process [19]. The reported OCT imaging results present structures that corresponded well with histological features of the wounded skin, suggesting the potential of OCT to visualize tissue regeneration and migration. However, these morphological studies did not contain vascular information.

Optical microangiography (OMAG), an extension of OCT, is a recently developed imaging technique capable of producing 3-D images of dynamic blood perfusion within microcirculatory tissue beds with an imaging depth of ∼2 mm [20], [21]. OMAG is a label-free technique, because it uses the intrinsic light scattering from flowing blood cells within patent vessels to produce the imaging contrast. An unprecedented sensitivity to the blood flow down to 4 µm/s was reported with the most recent development of ultrahigh sensitive OMAG [22], [23]. With such high sensitivity to slow flow velocities, the modality has been successfully employed to image the microcirculations within tissue beds of small animals and has also extended to human studies such as skin and eye [23]–[25]. _ENREF_6 Doppler optical microangiography (DOMAG), as a deviation of phase-resolved Doppler OCT technique [26], is capable of providing bidirectional blood flow velocity map [27]. By alternatively capturing data using OMAG and DOMAG scanning protocols, we demonstrated the capability of OMAG for volumetric and quantitative imaging of retinal blood flow in rats [28].

In the present work, we approach the feasibility of OMAG to track the 3-D tissue and vascular remodeling in a wound induced by a biopsy punch on the mouse pinna *in vivo*. We demonstrate for the first time that the OMAG system is capable of visualizing capillary networks (∼5 µm), detecting flow direction changes as well as determining both the vascular and corresponding tissue remodeling and growth due to a biopsy wound.

## System and Methods

### 1.1.Ethics statement

All experiments were performed on SKH-1E mice of 22–26 g weight. All procedures were performed with approval from the Institutional Animal Care and Use Committee at the University of Washington (protocol #4262-01).

### 1.2. OMAG system setup

**Figure 1:**
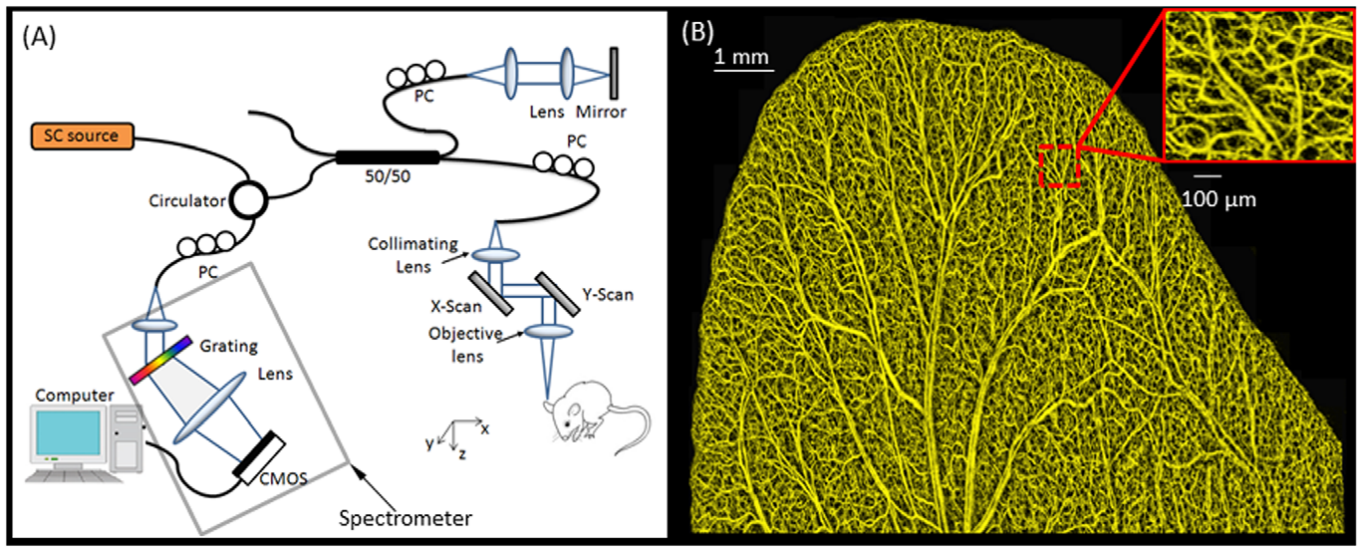
Schematic diagram of the spectral domain optical coherence tomography system and example of typical in vivo image obtained from the system. (a) SC source =  Supercontinuum light source; PC = polarization controller. (b) Projection-view OMAG image of the whole mouse pinna before the wound. The system was capable of resolving blood vessel networks down to the capillary level.

The system is illustrated in Fig.1 (A). The light emitted from a supercontinuum source (Koheras A/S, Denmark) was used as the light source to illuminate the system. The light source had a central wavelength ∼820 nm with a spectral bandwidth of ∼120 nm, providing ∼2.5 µm theoretical axial resolution in tissue for the system. The lights reflected from the reference mirror and backscattered from the sample were combined and formed interference signal that was then subsequently sent to a home-built spectrometer for detection. The spectrometer is based on a high speed complementary metal-oxide-semiconductor camera (4096 linear pixel-array, Basler SPL 4096-70 KM, Germany), giving an imaging speed of 70 kHz A-line rate for the system. At this imaging speed, the system sensitivity was measured at ∼90 dB around the zero delay line, which fell off to 70 dB at ±3 mm position. In the sample arm, we used a 10×objective lens with an effective focal length of 18 mm and achieved a lateral resolution of 5.8 µm as previously demonstrated [29]. The 3D imaging was accomplished by an x-y galvanometer scanner that scanned the probe beam in the sample arm at the sample surface.

### 1.3. OMAG imaging Protocol (OMAG)

To achieve OMAG, a specially designed scanning protocol was applied as previously described [30]. Briefly, in the fast scan (x) direction, 512 A-lines were captured to compose one B-scan cross sectional image. By setting a ∼2.4 µm spatial interval between every two A-lines, each B-scan image covered a range of ∼1.2 mm in the X-direction. In the y-scanning direction, 1000 B-frames were captured (5 frames were acquired at each position, rendering 200 total positions) to cover a range of ∼1.2 mm. With a 70 kHz A-line rate the frame rate of the system was 220 frames per second. The system required ∼9 seconds to finish one three dimensional data, which is short enough and suitable for *in vivo* experiments. We then applied the ultrahigh sensitive OMAG algorithm [23] to process the data and obtain both structural and flow image of the scanned volume. In this paper, the OMAG image was presented as a maximum projection view along the depth direction to facilitate the comparison and discussion below.

### 1.4. Doppler Optical Microangiography imaging Protocol (DOMAG)

DOMAG [27] was used to provide the blood flow velocity and flow direction inside vessels. In the fast scanning direction, 2000 A-lines were captured to compose one B-scan cross sectional image covering 1.2 mm. And in the slow scanning direction, 200 B-frames covering 1.2 mm were captured to form a 3D data volume. Before the ear punch, the camera speed was set to be 5 kHz, and the frame rate was 2 frames per second. Whereas, after the ear punch, due to decrease of the flow speed, we set the camera speed to be 2 kHz and the frame rate is 0.8 frames per second because of the reduction of blood flow at this time. The axial flow velocity can be derived from the phase difference between adjacent A-lines within one B-frame, which is introduced by the motion of blood cells using the following equation:



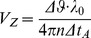
(1)where λ_0_ is the central wavelength of the light source (820 nm), n is the refractive index of the tissue (∼1.35) and Δt_A_ is the time interval between adjacent lines. To determine the absolute blood flow velocity, the angle between the flow velocity vector and the vector of incident OCT light, also known as the Doppler angle was determined using the three dimensional data set captured using OMAG [28].

### 1.5. Animal model for wound induction

To determine whether OMAG could noninvasively track the vascular architecture, blood flow and structural changes within the tissue during the wound healing process, four SKH1-E mice (22 to 26 g) (Charles River, Holllister, CA) were imaged at various time points after a wound was induced with a biopsy punch. A separate animal was used to image 1) the whole pinna, 2) the blood flow direction during the acute response, 3) capillary recruitment during the acute response and 4) repeated imaging of structure and vasculature form 30 min to 60 days. Animals were subjected to a 12 h light and dark cycle, and allowed to drink and eat *ad libitum*.

Mice were anesthetized with isoflurane (0.2 L/min oxygen and 0.8 L/min air) by a face mask. The pinna was immobilized with double-sided tape onto a glass slide to minimize motion artifacts. As a control, the mouse pinna was imaged prior to the wound. To induce a wound in the ear, a 0.5 mm size biopsy punch was sterilized, placed on the dorsal side of the pinna and used to punch a round hole through the skin. The bleeding was washed out with medical grade saline. After onset of the wound, OMAG imaging was obtained from the same regions as the control image. In order to observe the acute response of blood flow due to the wound injury, a series of OMAG images were obtained on one animal at 5, 10, 20 and 30 minutes after the wound. Also, OMAG images were obtained at 30 minutes and 4, 8,12,18,22,26,32,46 and 60 days on the same animal after the biopsy punch to monitor the chronic wound healing process.

The average and standard deviation epidermal thickness was quantified. The epidermal thickness from each time point was normalized to the control data point.

## Results

The OMAG system depicted in Figure 1a was used to image the blood vessel network of a mouse pinna. An OMAG image before the wound is presented in Figure 1b. Given that the OMAG algorithm for microcirculation imaging used in these studies is sensitive to slow flow velocities, both large and small vessels can be visualized. In addition, as shown by the inset, the dense capillary network is well defined.

**Figure 2:**
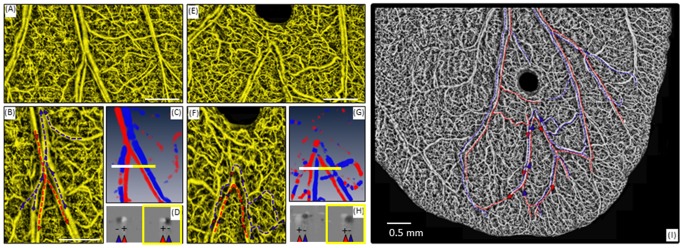
Determination of blood flow direction using DOMAG after induction of the wound on the mouse pinna. Projection view of the OMAG vascular map (A) before and (E) approximately 3 min after the wound. Flow direction within an artery and vein (B) before and (F) after the wound. The direction of arterial and venous flow is indicated by the red and blue dashed lines and arrows. (C) 3D color map of the axial flow velocity obtained with DOMAG before the wound. Blue indicates −1 mm/s and red indicates +1 mm/s. (G) 3D color map of the axial flow velocity obtained with DOMAG after the wound Blue indicates −0.4 mm/s and red indicates +0.4 mm/s. (D) 2D cross-sectional grey phase Doppler image of the axial flow velocity at the lines in (C). The grey scale velocity range is black −1 mm/s to white +1 mm/s. (H) 2D cross-sectional grey phase Doppler image of the axial flow velocity at the lines in (G). The grey scale velocity range is black −0.4 mm/s to white +0.4 mm/s. After the artery and vein branch, the vessels from the left branch (white box) show a reverse in flow direction, as opposed to the vessels from the right branch (yellow boxes) (I) Image of the whole ear indicating the flow direction after the wound. Red dashed line indicates arterial flow and blue dashed line indicates venous flow.

To determine the changes in blood flow velocity and direction, we imaged a small area of the mouse pinna. Figure 2A and 2E show the OMAG microangiography of the mouse pinna before and after (3 minutes) the wound. With DOMAG we calculated the blood flow velocity and direction. The three-dimensional color map for the direction of flow in four main vessels is shown in Figures 2C and 2G, where red and blue indicate flow against (arterial) and towards (venous) the incident OCT beam, respectively. The cross-section at the location of the white and yellow lines in Figures 2C and 2G are presented in Figures 2D and 2H, respectively, which presents the Doppler phase change image in the axial direction. The vessel directionality is indicated with the red (+) and blue (−) arrows, and represent downward and upward flow direction, respectively. The wound severed both arterial and venous vessels, and rapidly induced a reversal in blood flow direction in a branch of the downstream circulation (white box in Figure 2D and 2H). The other branch (yellow box in Figure 2D and 2H) maintained an intact flow direction. It is known that the arteries have smaller vessel diameters than the veins, and that the blood flow direction of the arteries is toward, while the vein is away from the edges of the pinna. The dashed arrows in Figures 2B and 2F indicate the flow direction from the designated arteries (red) and veins (blue). The main artery branches into two smaller vessels. Likewise, two smaller vessels converge into one larger vein. The comprehensive image (Figure 2I) presents a larger view of the blood flow after the wound, which caused a redirection of the flow. This figure helps to understand how the blood flow of the whole ear responds to supply the wound area after the injury. By using the three-dimensional information of the vessels presented in Figures 2B and 2F, we estimated the Doppler angle which allowed us to calculate the total blood flow inside the vessels of interest. As an example of DOMAG's ability to quantify blood flow, we determined the absolute flow velocity of the artery (white box in Figure 2D) to be reduced after the wound from ∼6 mm/s to 1.5 mm/s.

**Figure 3:**
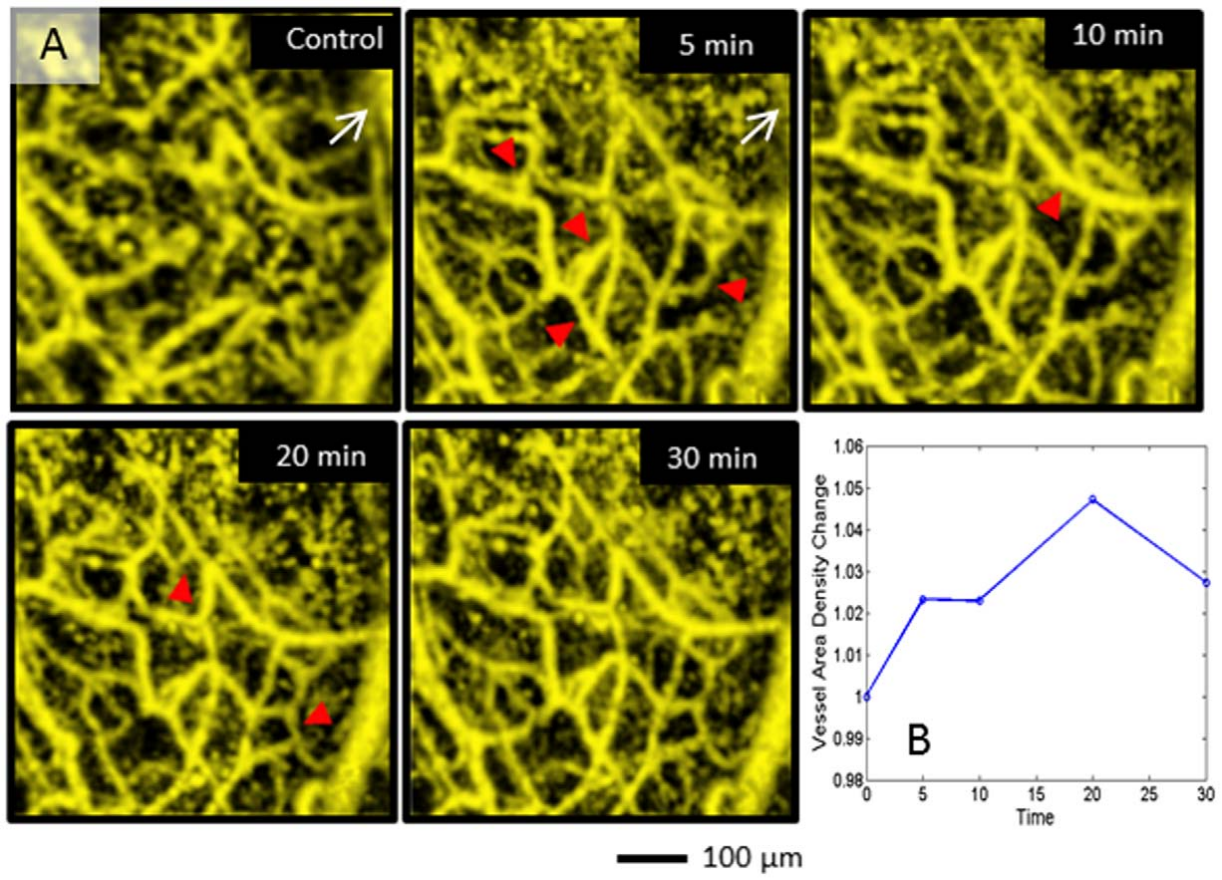
Capillary recruitment of peripheral vessels at several time points after the wound. (A) White arrow indicates the large vessels that disappear, and the red arrows indicate the capillary vessels that are recruited after the wound. (B) Relative change in vessel area density in response to biopsy punch.

To understand the acute blood vessel responses after the biopsy punch, OMAG images were captured at 5, 10, 20 and 30 minutes after induction of the wound (Figure 3). The wound was located in the upper right hand corner of the field of view. This can be visualized by the loss of blood flow and thus disappearance of the vessel depicted by the white arrow. Blood flow in pre-existing capillaries was immediately recruited after initiation of the wound (red arrows), and its domino effect could be observed as other capillary branches appeared at 10 min. By 20 min more vessels are detected compared with 10 min indicating increased compensatory blood flow (red arrows). To quantify the capillary recruitment, the relative vessel area density change was obtained as described by Reif et al. [31]. The vessel area density increased by 5 min and peaked at 20 min after injury, suggesting an increase in the number of capillaries present after the injury.

**Figure 4:**
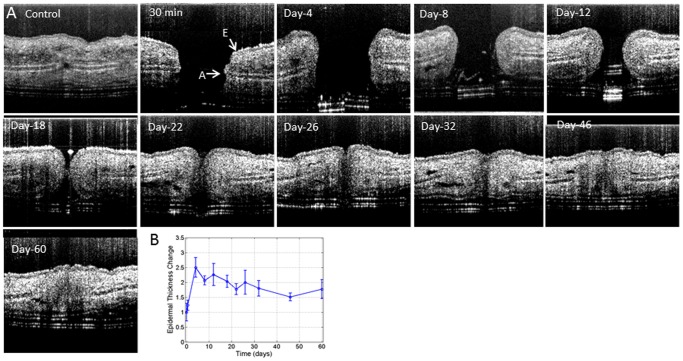
OMAG cross-sectional structural images of the mouse pinna *in vivo* at several time points. (A) The image size is 1.2 mm×1.2 mm. A, auricular cartilage, E, Epidermis. The epidermal layer increased in thickness and migrated together to from a closure, but the auricular cartilage never fully recovered at 60 days post-injury. (B) Quantification of relative change in epidermal thickness over time.

**Figure 5:**
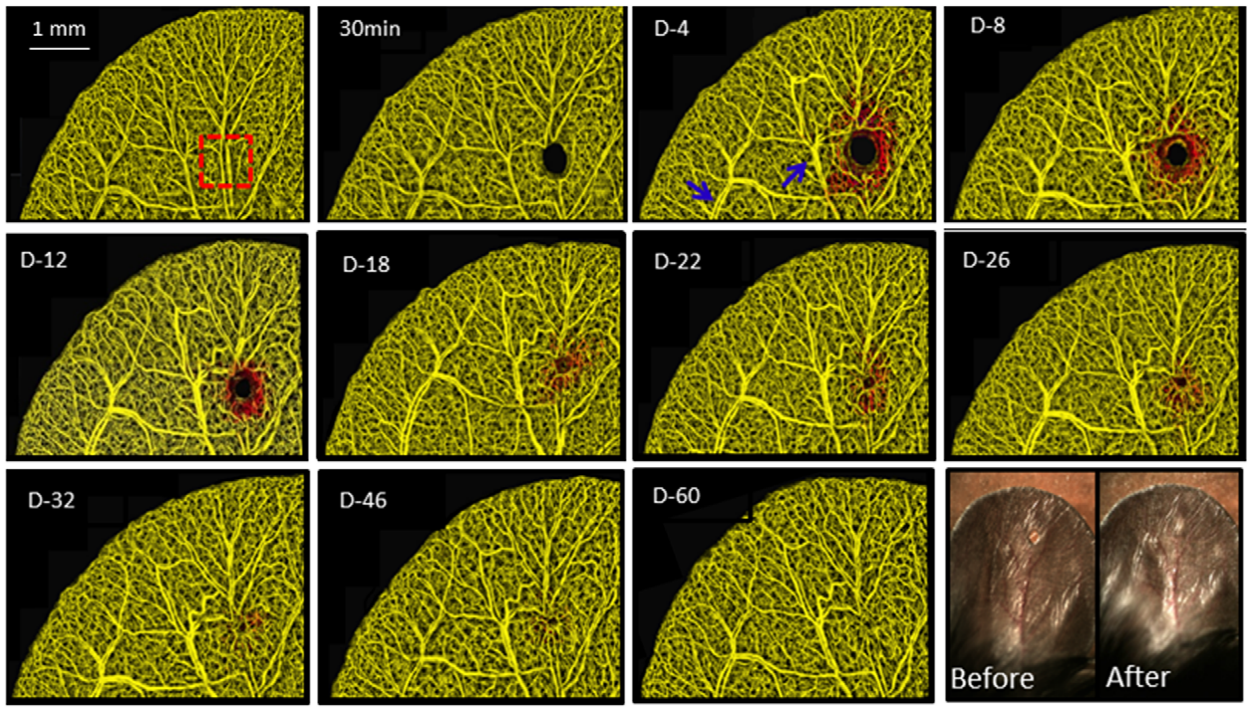
Projection view OMAG images of the pinna before and after wound induced by biopsy punch *in vivo* at different time points. The red color indicates vasculature within thicker tissue compared to the baseline control. Compared to baseline, progressive vessel regulation (vessels pointed by blue arrows) and neovascularization observed as red looped vessels surrounding the wound develop at 4 days after punch biopsy was induced. In the lower right corner, a light photograph of the wound at days 4 and 60.

**Figure 6:**
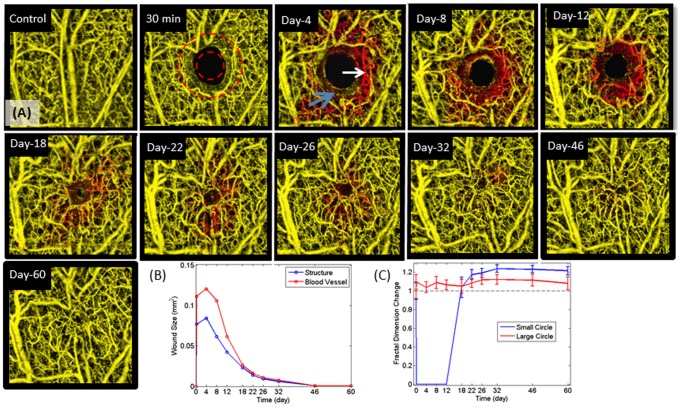
Depth-resolved projection view and corresponding changes in wound size and fractal dimension over time. (A) Projection view of the OMAG wound vasculature for the different time points in Figure 5 for a small field of view (1.2 mm×1.2 mm). (B) Size of the wound at different time points. These were calculated by the size of the structure wound (white arrow in day 4) and blood vessel wound (blue arrow in day 4). (C) Mean and standard deviation of the change in the fractal dimension value calculated at the periphery of a small and large circle around the wound (indicated in the 30 min image).

Structure images of a cross-section of the pinna obtained with the OCT system is shown in Figure 4. The location of the epidermis (E) and auricular cartilage (A) is indicated by corresponding arrows. By day 4 the edges of the wound are raised compared with the control image. The epidermal thickness (Fig.4B) in the structural images drastically increases at day 4 and gradually subsides thereafter, although never returning to the pre-injury thickness within the timeframe investigated (60 days in this study). To determine the behavior of blood vessel development and structural wound closure during wound healing, a series of OMAG images (Figs. 5 and 6) were obtained on the same animal before and at 30 min and 4, 8, 12, 18, 22, 26, 32, 46 and 60 days after the biopsy punch was performed on the mouse pinna. The branches of an adjacent artery and vein around the wound became gradually thicker starting from day 4 up to day 26 (blue arrow in Figure 5) suggesting a hyperemic response to the tissue surrounding ischemic area.

To correlate observed changes in structural thickness with vascular development, the data was processed as a depth-resolved image. A line (data not shown) was assigned to the highest point on the control image. Any vessel structure below this line is depicted in a yellow color and any vessel structure higher than this line is designated as a red color. Therefore the red color in Figures 5 and 6 represents an increased structural thickness when compared with the baseline image. As wound healing progresses, the thickness gradually returns to baseline levels. In the lower right hand panel of Fig 5 a picture of the pinna was taken at day 4 and 60. After 60 days no wound is visible by eye. However, the microangiography in Figures 5 and 6 clearly shows that the vasculature is abnormal and the structural image in Figure 4 shows the cartilage never completely unites by day 60.

The high resolution achieved by our system enabled us to visualize morphological details. Figure 6 shows enlarged microangiography images of the wound area corresponding to the positions marked by the dashed-line red square and biopsy punch over 30 min to 60 days in Figure 5. After the wound induction, the tissue and blood flow was totally absent as shown by the black holes in Figures 5 and 6. Because OMAG only renders images from which there is blood flow, lack of flow appeared as a black space. Figure 6 shows the formation of functional vessels along with the increased thickness of the pinna around the area of the wound by day 4 (white arrow). This growth of new vasculature is coiled and torturous in nature suggesting angiogenesis. By day 18, numerous capillaries are present within newly generated tissue.

Figure 6B shows the area of the wound size, which was quantified for both the structure (Figure 4) and blood vessel images (Figure 6A). New tissue formation occurred at a high rate between days 12 to day 22 as shown by the decreased wounds structure size as new tissue is generated and cells migrate toward each other. We also estimated the wound size based on the blood vessel images. The wound size estimated by the blood vessel image reaches a peak of ∼0.11 mm^2^ compared to ∼0.075 mm^2^ estimated by the structure image. The blood vessel wound size is larger than the structure wound size because there was no blood flow surrounding the wound 30 min after the biopsy punch. In figure 6A this is most apparent at day 4 where the edge of the wound is visible and the absence of vessels is apparent (blue arrow).

Fractal dimension was used to quantify the blood vessel tortuosity during the wound healing process [31]. Briefly, the vessels were segmented and a binary image was created and skeletonized. The fractal dimension was calculated over the skeletonized image within a small window (∼100 µm x100 µm). Two circles (a small and a large) centered at the wound were created as shown in Fig 6 at 30 min. The mean and standard deviation of the fractal dimension at the periphery of the circle was calculated for each timepoint (Figure 6C). In the small circle, the fractal dimension disappears after the wound (as expected given that there are no vessels present). The vessels reappear and stabilize at a fractal dimension value 20% higher than the baseline at 60 days. This indicates that the new vessels formed are more tortuous as typically observed in angiogenic vessel formation. The vessels in the large circle have a ∼10% increase in fractal dimension after the wound. This value remains fairly constant throughout time. The increase is possibly due to the recruitment of existing capillary vessels.

## Discussion

OMAG allowed the non-invasive visualization and quantification of vascular and structural changes *in vivo* over time after a biopsy-induced wound on the mouse pinna. This study presents three major findings. First, OMAG had the sensitivity to detect the acute and chronic changes in the complex microvascular angiography in the skin after induction of the wound. Second, DOMAG was able to determine the acute changes in blood flow velocity and direction which resulted after the normal vasculature was disturbed. Third, OMAG was able to detect the increased thickness of the skin by depth-resolved imaging and determine the rate of wound healing with the corresponding increases in fractal dimension.

For the flow velocity and direction analysis before and after the wound, we used conventional DOMAG method which calculates the phase change between adjacent A-lines within one B-scan rather than calculate the phase change between A-lines within inter B-scans in ultrahigh sensitive DOMAG. This is because the axial velocity range could be unambiguously measured by the ultrahigh sensitive DOMAG without phase wrapping is −110 µm/s <V_z_<110 µm/s, which could not detect the flow speed and determine the direction correctly of arteries and veins in the mouse pinna. The A-line rate we used for data acquisition is 5 KHz and the data was processed using the DOMAG algorithm as previously described [27]. This scanning speed provides a detectable axial velocity range that could be unambiguously measured was −1 mm/s <Vz<1 mm/s, the directional flow from the artery and vein could be effectively measured as shown in Figure 2. However, after the wound, the flow speed in the downward artery and vein become very slow relative to baseline. To measure the blood flow velocities and visualize the flow direction after the wound, we needed to use a longer time interval between subsequent A-lines to increase the detection sensitivity. Hence, the pinna was imaged at a 2 kHz A-line rate and detectable axial velocity range was −0.4 mm/s <Vz<0.4 mm/s so the artery and vein were effectively measured and separated.

We observed changes in capillary responses 5–30 min after the wound (Figure 3) indicating that OMAG possesses the sensitivity to detect capillary recruitment. Although we did not directly measure blood flow velocity directly in these early time points the sensitivity of the system was capable of detecting capillary vessels. We calculated the relative vessel area density change as a means of quantifying capillary recruitment. In Figure 3, capillary recruitment appears as early as 5 min after the wound. Since angiogenesis cannot occur in the time period of 5–30 min, the appearance of capillaries is due to increased blood flow from preexisting capillaries. This capillary recruitment is likely related to external stress such as changes in blood flow, velocity and tissue oxygenation after the wound eliminated some of the pre-existing vessels [32]. The vasculature redirects blood flow through collateral circulation as a possible compensatory mechanism to provide blood supply to ischemic and/or damaged tissue.

The wound healing process is comprised of distinct yet overlapping phases involving inflammation, tissue formation and tissue remodeling [1]. Inflammation is a protective attempt by the organism to remove the injurious stimuli and to initiate the healing process [33]. Inflammation causes redness swelling which may account for the increased thickness detected from both the structure and depth resolved image processing. Additionally, new tissue formation is initiated by the thickening of the epidermis immediately adjacent to the wound where the epithelial cells are proliferating and migrating to close the wound. This is in agreement with Cobb MJ et al. [34], who also observed that the re-epithelialized wound was slightly higher than the surrounding surface. Since tissue requires a functional vasculature for survival, the development of new vasculature should be concomitant with the development of new tissue. We were able to correlate the increased thickness with the development of new vasculature (Figures 4, 5 and 6).

Although several animal models of wound healing have been reported [35], closed biopsy punch on the mouse pinna is becoming an increasingly utilized method for investigating the wound healing process by optical imaging [36] [14] [37] [34]. The pinna is easily optically imaged compared with complications encountered with traditional chamber [38]or subcutaneous models of wound healing [39] and allows for high repeatability for imaging the same area over time. This model differs from full thickness skin removal on either the dorsal and or tail [40] in that there is no underlying muscle as a scaffold for the generation of new tissue and blood vessels and the vascular and tissue changes during wound healing may be different than standard models of cutaneous wounds. However, the phases of inflammation, tissue formation and remodeling appear universal among the models. Rege et al [14] used laser speckle imaging to study the microvascular flow that accompanied angiogenic changes in vascular architecture during wound healing angiogenesis using a 2 mm biopsy punch on the mouse ear over the three phases of wound healing which were validated with histology.Angiogenesis is the physiological healing process involving the growth of new blood vessel from pre-existing vessels [41]. Angiogenesis is a normal and vital process in growth and development, as well as in wound healing. During the time of wound closure, days 4–60, numerous capillaries appeared within newly generated tissue within the wound. We analyzed the fractal dimension change within the wound and found an increase during the tissue formation. Previous reports describe newly developing blood vessels extend to form a short sprouts whose tips move in the direction of the wound center. As sprouting occurs at the capillary tip, it migrates and leaves a new capillary that is contiguous with the parent vessel. While the tipped blood vessels are traveling toward the wound center there are many tip to tip and tip to vessel contacts in which, two tipped vessels join to form a single loop, or arcade, in a process termed anastomosis [42], which can be observed in Figures 5 and 6 as the coiled and torturous new vasculature also depicted in red on days 4–26.

## Conclusion

We demonstrate the ability of non-invasively tracking *in vivo* the dynamic behavior of the structural and vasculature components of a mouse pinna, after inducing a wound, using OMAG. The high resolution achieved by the system enabled us to visualize morphological and microvascular details during the healing process. To design effective treatments, a more comprehensive understanding of tissue and vascular restoration during wound healing is needed. Our data supports OMAG as a practical tool for tracking tissue formation and its vascular remodeling after an injury. We anticipate the use of OMAG for both animal studies and human studies to investigate repair strategies and the novel therapeutics that are designed to facilitate repair for wound healing and other skin diseases.
